# Anodal Transcranial Direct Current Stimulation (tDCS) Over the Right Primary Motor Cortex (M1) Impairs Implicit Motor Sequence Learning of the Ipsilateral Hand

**DOI:** 10.3389/fnhum.2018.00289

**Published:** 2018-07-19

**Authors:** Ariane Keitel, Henning Øfsteng, Vanessa Krause, Bettina Pollok

**Affiliations:** Medical Faculty, Institute of Clinical Neuroscience and Medical Psychology, Heinrich Heine University Düsseldorf, Düsseldorf, Germany

**Keywords:** acquisition, ipsilateral, motor training, serial reaction time task (SRTT), excitability, non-invasive brain stimulation

## Abstract

Motor sequence learning is associated with the activation of bilateral primary motor cortices (M1). While previous data support the hypothesis that the contralateral M1 is causally involved in the acquisition as well as early consolidation of a motor sequence, the functional significance of the ipsilateral M1 has yet to be solved. Transcranial direct current stimulation (tDCS) allows the non-invasive modulation of cortical excitability. Anodal tDCS applied to the left M1 has been shown to facilitate implicit motor sequence learning of the right hand most likely due to increased excitability. The present study aims at characterizing the functional contribution of the ipsilateral (right) M1 on implicit motor sequence learning of the right hand. To this end, 24 healthy, right-handed subjects received anodal and sham tDCS to the right M1 in a counterbalanced order. Stimulation started 8 min prior to training on a variant of the serial reaction time task (SRTT) with the right hand and persists over the entire training period. The SRTT comprised a fixed eight-digit sequence. A random pattern served as control condition. Reaction times were assessed before and at the end of the acquisition (EoA) immediately after training on the SRTT. The analysis revealed significantly faster reaction times of both hands independent of tDCS condition in sequential trials. However, the gain of reaction times was significantly smaller following anodal as compared to sham tDCS. The data suggest that anodal tDCS applied to the right M1 impairs implicit motor sequence learning of both hands. The underlying mechanism likely involves alterations of the interaction between bilateral M1.

## Introduction

Complex movements are often sequenced and therefore motor sequence learning facilitates a variety of daily activities. The serial reaction time task (SRTT) is a well-established tool for the induction and assessment of implicit motor sequence learning (Nissen and Bullemer, 1987). It is characterized by the acquisition of a motor sequence through its repetition resulting in faster reaction times and more accurate motor performance as practice proceeds (for reviews see Doyon, 2008; Dayan and Cohen, 2011). Typically, the subjects are not aware of the underlying sequential pattern indicating implicit learning, which is mediated by a cortico-striatal-cerebellar network (Doyon and Benali, 2005). Within this network, the primary motor cortex (M1) plays a crucial role for the acquisition and early consolidation of a motor sequence (Pascual-Leone et al., 1994; Karni et al., 1995; Muellbacher et al., 2002; Ungerleider et al., 2002; Antal et al., 2004; Lu and Ashe, 2005; Rosenthal et al., 2009; Wilkinson et al., 2010).

Learning of a motor skill with one hand is often associated with a performance gain of the contralateral, untrained hand (Halsband, 1992; Thut et al., 1996; Grafton et al., 2002; Perez et al., 2007a,b; Lee et al., 2010; Hinder et al., 2013a,b) suggesting the involvement of the hemisphere ipsilateral to the trained hand. Indeed, neuroimaging and non-invasive brain stimulation (NIBS) studies point toward the involvement of bilateral M1 in motor learning (Chen et al., 1997; Davare et al., 2007; Perez et al., 2007b; Duque et al., 2008; Lee et al., 2010).

Transcranial direct current stimulation (tDCS) is a NIBS technique that allows the modulation of cortical excitability along with behavioral performance in a polarity-dependent manner (for a review see Madhavan and Shah, 2012). Although the exact mechanisms are not entirely understood, tDCS has been shown to alter the resting membrane potential thereby changing the excitability of the stimulated area (for reviews see Stagg and Nitsche, 2011; Shin et al., 2015). While anodal tDCS has been found to increase motor-cortical excitability, cathodal tDCS has been related to its reduction.

Anodal tDCS applied to the left M1 facilitates implicit motor sequence learning of the contralateral right hand (Nitsche et al., 2003b; Kang and Paik, 2011), whereas effects of cathodal tDCS are often less consistent (e.g., Nitsche et al., 2003b). Performance changes of the ipsilateral hand following NIBS have been found as well. Kobayashi and co-workers (Kobayashi et al., 2004, 2009) showed that reducing M1 excitability by means of 1 Hz repetitive transcranial magnetic stimulation (rTMS) facilitates motor learning of the ipsilateral hand while performance of the contralateral hand was reduced. These data suggest that rTMS over M1 of one hemisphere may affect the excitability of the contralateral homolog and supports the hypothesis of interhemispheric rivalry.

The present study aims at testing the hypothesis that increasing the excitability of the M1 ipsilateral to the trained hand by means of anodal tDCS inhibits motor sequence learning of the right hand.

## Materials and Methods

### Subjects

Twenty-four healthy subjects (9 male) with a mean age of 27.08 ± 1.23 years [mean ± standard error of the mean (SEM)] participated in the present study. All subjects were right-handed according to the Edinburgh Handedness Inventory (Oldfield, 1971). General exclusion criteria comprised history or family history of epileptic seizures, migraine or other neurological or psychiatric disorders, intake of central nervous system affecting drugs, cardiac or brain pacemaker and pregnancy.

### Experimental Design and Procedure

All subjects received anodal vs. sham tDCS in a counterbalanced order in two sessions separated by at least 1 week in order to avoid carry-over effects. Subjects were naïve regarding the exact aim of the study and the respective stimulation condition. Blinding of the main investigator regarding the DC-condition was achieved by a second investigator handling the DC-stimulator only.

### Serial Reaction Time Task

A version of the well-established SRTT (Nissen and Bullemer, 1987) was employed in order to induce implicit motor sequence learning. Subjects were introduced to the SRTT as a simple reaction time task. During the SRTT four horizontally aligned bars were presented on a screen. Each bar corresponded to one of four response keys of a custom-made button-box. The participants were instructed to respond correctly as fast as possible as soon as one of the four bars changed its color from dark blue to light blue by button press. Responses were given with the index (1), middle (2), ring (3), or little finger (4). The button-box was connected to a standard Windows PC. While performing the task, subjects were comfortably seated in a reclining chair. The screen was positioned with a distance of 2.66 m in front of the subjects and the stimuli were presented with a visual angle of 12.78°. The correct response triggered the color change of the next bar after a fixed inter-stimulus interval of 1,000 ms. In case subjects failed to press the correct button, the bar remained light blue until the correct response was chosen. Stimuli were presented in a sequential pattern with a fixed repeating eight-digit sequence as well as in a randomly varying pattern with the constraint that in both patterns each stimulus appeared with the same frequency. The presence of the sequence was unknown to the subjects. In order to avoid training effects between the two sessions, two parallel versions of the SRTT were employed (sequence 1: 3-2-1-4-3-2-4-1; sequence 2: 2-3-4-1-4-1-2-3). Prior to the training both patterns were presented twice and reaction times of the left and the right hand were determined subsequently. The sequential pattern for the left hand was presented as the mirror image of the right-hand sequence requiring homologous finger movements to those on right-hand trials. For baseline measurement the order of tasks (random vs. sequential) and hand (left vs. right) was counterbalanced across subjects and conditions.

**FIGURE 1:**
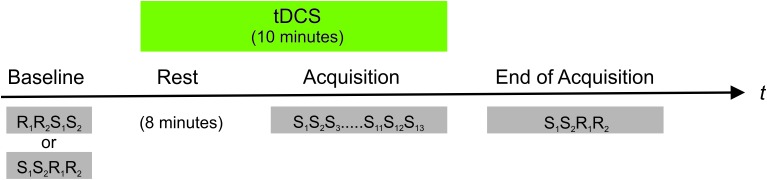
Overview of the experimental procedure. In a counterbalanced order anodal vs. sham tDCS was applied to the right (ipsilateral) M1 starting 8 min prior to and persisting during training on a SRTT. The SRTT comprised 13 repetitions of an eight-digit fixed sequence (S). An eight-digit randomly varying pattern (R) served as control condition. The subjects were trained on the SRTT with their right hand only. Reaction times of both hands were measured before (baseline) and after training on the SRTT (end of acquisition). In baseline trials the order of random and sequential trials were counterbalanced across subjects and tDCS conditions (i.e., anodal vs. sham). After training on the SRTT, sequential trials always preceded random runs.

After baseline performance was determined, subjects were trained on the SRTT with the right hand, only. The SRTT comprised 13 repetitions of the eight-digit sequence requiring a total of 104 button presses. Although the number of sequence repetitions is comparatively low, it was chosen as it has successfully elicited implicit motor learning in previous studies by our own group (Krause et al., 2016; Focke et al., 2017) and by others (Kang and Paik, 2011). Immediately after the training reaction times of both hands were measured [end of acquisition (EoA)] always beginning with the sequential pattern, followed by a random pattern and always starting with the right hand. The order of SRTT versions and tDCS conditions (anodal vs. sham tDCS) was counterbalanced across sessions and subjects. For an overview of the experimental procedure see **Figure 1**.

### Localization of M1

In order to determine the right M1 hot-spot, motor-evoked potentials of the left fore-arm muscles were elicited by single TMS pulses delivered with a standard figure-of-eight coil (MC-B70, MagPro Stimulator, MagVenture, Willich, Germany). The coil was held tangentially to the scalp with the handle pointing backward and laterally at an angle of about 45° away from the midline. The coil was positioned over the M1 hand area to elicit motor responses. By moving the coil in 0.5 cm steps anterior, posterior, medial, and lateral to this area, the exact localization of the area which evoked the maximal motor response was determined. This location was marked on the scalp with a pen and used for positioning of the tDCS electrode.

### Transcranial Direct Current Stimulation

Transcranial direct current stimulation was applied via two saline-soaked sponge electrodes connected to a DC-stimulator (DC-Stimulator Plus, Eldith, Neuroconn, Ilmenau, Germany). In order to decrease skin resistance, the skin was cleaned and slightly abraded prior to stimulation. The stimulation electrode (3 cm × 3 cm) serving as anode was placed over the right M1. The return electrode (5 cm × 5 cm) was placed above the left orbita. The smaller electrode over M1 in combination with a larger return electrode has been shown to increase the focality of tDCS over the stimulated area (Nitsche et al., 2007). TDCS was applied with an intensity of 0.25 mA (0.0278 mA/cm^2^ current density under the stimulation electrode and 0.01 mA/cm^2^ under the reference electrode). Stimulation started 8 min prior to training on the SRTT and continued over the entire course of training. TDCS was terminated manually immediately after training had been completed. Training on the SRTT lasted on average for 2.28 ± 0.16 min (mean ± SEM) resulting in 10.28 ± 0.16 min of stimulation on average. Additionally, 10 s of fade-in time was applied. Impedance was kept below 10 kOhm. For sham stimulation, tDCS was applied for 30 s with additional 10 s of fade-in and fade-out time. The stimulation procedure was in accordance with current safety guidelines (Nitsche et al., 2003a).

The subjects and the investigator were blinded regarding the actual stimulation condition (anodal vs. sham tDCS). In order to assess whether blinding was successful, the subjects were asked to rate what stimulation type they had received as well as to evaluate the confidence of their decision at the end of each session using a numerical rating scale ranging from 1 (completely uncertain) to 10 (completely certain). Anodal tDCS was identified correctly in 37.5% of anodal sessions with a mean subjective confidence of 5.44 ± 0.90 (SEM) and sham stimulation was identified correctly in 58.3% of sham sessions with a mean confidence of 6.32 ± 0.69 (SEM). Since the subjects’ stimulation ratings were around chance level, blinding appears to be adequate. In order to determine whether learning might have become explicit, subjects were asked at each session’s end whether they had detected a pattern within the SRTT. One subject was able to reproduce both and three subjects were able to reproduce one of the two sequences correctly indicating that implicit learning took place in the majority of subjects.

### Ethics

All subjects gave their informed written consent prior to study participation. The study was approved by the local ethics committee of the Medical Faculty, Heinrich-Heine-University, Duesseldorf, Germany (study no. 3347, amendment 2014) and was in accordance with the declaration of Helsinki.

### Data Analysis

Reaction times were determined as the temporal distance between stimulus onset and button press. The first three button presses of either hand were excluded from further analysis in order to account for the subjects’ initial familiarization with the task. Values outside confidence intervals within individual and group data (mean ±2 standard deviations) were classified as outliers and discarded from the analysis. 7.55% of individual and 3.82% of group data were removed according to these criteria.

Normal distribution of the data was confirmed using Kolmogorov-Smirnov goodness-of-fit test. Repeated measures analyses of variance (ANOVA) with factors *stimulation* (anodal vs. sham tDCS), *time* (baseline vs. EoA), *task* (random vs. sequential), and *hand* (right vs. left) were computed. In case of violation of sphericity, Greenhouse-Geisser corrections were applied. Paired *t*-tests were utilized for *post hoc* analyses. Bonferroni correction was applied for multiple comparisons. Statistical analyses were performed using IBM SPSS Statistics 22 (SPSS, Chicago, IL, United States).

## Results

The ANOVA yielded a significant main effect of *hand* [*F*_(1,23)_ = 15.17, *p* = 0.001], *time* [*F*_(1,23)_ = 86.13, *p* < 0.001] and *task* [*F*_(1,23)_ = 48.18, *p* < 0.001] but no significant main effect of *stimulation* [*F*_(1,23)_ = 0.12, *p* = 0.73]. In addition a significant *time* ×*task* interaction [*F*_(1,23)_ = 8.80, *p* = 0.007] was found, whereas the *stimulation* ×*time* interaction just missed significance [*F*_(1,23)_ = 4.09, *p* = 0.055]. Moreover, a significant three-way interaction between *stimulation*, *time*, and *task* [*F*_(1,23)_ = 5.67, *p* = 0.026] was revealed. No further significant interactions were observed (all *p* > 0.14). In order to disentangle the three-way interaction, *post hoc* ANOVAs were performed with factors *stimulation* (anodal vs. sham tDCS) and *time* (baseline vs. EoA) separately for sequential and random trials.

**FIGURE 2:**
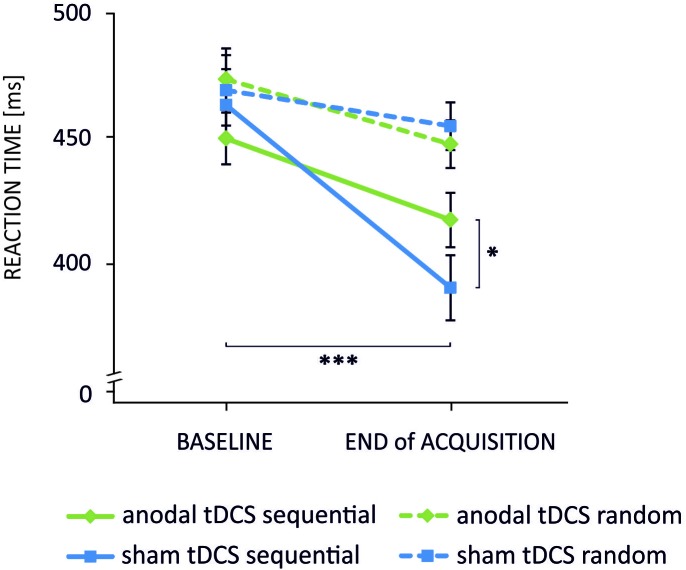
Mean reaction times indicating the *stimulation×time×task* interaction. Reaction times decreased significantly from baseline to end of acquisition in sequential trials following anodal (green solid line) and sham (blue solid line) tDCS. Noteworthy, the decrease was significantly smaller in the anodal condition. Reaction times of the random pattern decreased also over time but were not differentially modulated by tDCS (^∗∗∗^*p* < 0.001; ^∗^*p* < 0.05). Error bars indicate the standard error of the mean (SEM).

The analysis of reaction times in sequential trials yielded a significant main effect of *time* [*F*_(1,23)_ = 65.69, *p* < 0.001] but not of *stimulation* [*F*_(1,23)_ = 0.34, *p* = 0.57]. The *stimulation*×*time* interaction was found to be significant [*F*_(1,23)_ = 11.14, *p* = 0.003]. *Post hoc t*-tests revealed that reaction times decreased significantly from baseline to EoA indicating a performance gain over time in both tDCS conditions [anodal tDCS: baseline vs. EoA: *t*_(23)_ = 4.26, *p* < 0.001; sham: baseline vs. EoA: *t*_(23)_ = 7.30, *p* < 0.001]. However, at EoA reaction times were significantly faster following sham as compared to anodal tDCS [*t*_(23)_ = 2.16, *p* = 0.042]. ANOVA for random trials revealed a significant main effect of *time* [*F*_(1,23)_ = 8.24, *p* = 0.009] indicating that reaction times decreased from baseline to EoA. Neither a significant *stimulation*×*time* interaction [*F*_(1,23)_ = 0.70, *p* = 0.41] nor a significant main effect of *stimulation* [*F*_(1,23)_ = 0.055, *p* = 0.82] was observed. The results are summarized in **Figure 2** and **Table 1**.

**Table 1:** Mean reaction times ( ± standard error of the mean (SEM)) at Baseline and End of Acquisition (EoA) during anodal and sham tDCS for the right and left hand as well as averaged across both hands.

	Anodal tDCS				Sham tDCS			
	Sequential		Random		Sequential		Random	
	Baseline	EoA	Baseline	EoA	Baseline	EoA	Baseline	EoA
Right hand	439.24 ± 12.30	401.04 ± 13.39	461.64 ± 14.23	443.41 ± 10.93	455.99 ± 14.65	372.80 ± 10.43	468.49 ± 16.54	445.67 ± 12.31
Left hand	460.37 ± 10.81	433.27 ± 11.60	485.39 ± 11.39	451.58 ± 10.40	470.56 ± 15.52	407.18 ± 17.18	469.71 ± 14.53	463.84 ± 10.26
Mean	449.80 ± 10.37	417.15 ± 10.93	473.52 ± 12.36	447.50 ± 9.53	463.27 ± 14.36	389.99 ± 13.04	469.10 ± 14.13	454.76 ± 9.56


## Discussion

The aim of the present study was to investigate whether anodal tDCS applied to the right M1 affects implicit motor sequence learning of the ipsilateral right hand. The data were compared with those from the untrained left hand. The analysis revealed that reaction times of each hand were not distinctively modulated by training on the task or tDCS. More precisely, although the SRTT was trained with the right hand, only, reaction times of both hands were significantly faster at EoA as compared to baseline. As the main finding, the analysis suggests that anodal tDCS attenuated performance gain obtained after training. This pattern was restricted to sequential trials indicating a specific effect on motor sequence learning rather than on reaction times in general.

Faster reaction times at EoA as compared to baseline in sequential trials are indicative of implicit motor sequence learning. Noteworthy, faster reaction times occurred in random trials as well, but this effect was smaller than that observed in the sequential trials. Comparable effects have been reported in previous studies (Grafton et al., 2002; Perez et al., 2007b) and are likely due to familiarization with the task.

Both hands showed comparable performance patterns, which is reflected by the absence of a significant interaction involving the factor *hand*. This result suggests that the left hand takes advantage of right-hand training, a finding that agrees well with previous studies showing that a new motor skill acquired with one hand leads to facilitation of performance of the contralateral, untrained hand (Halsband, 1992; Thut et al., 1996; Grafton et al., 2002; Perez et al., 2007a,b; Lee et al., 2010; Hinder et al., 2013a,b).

Previous data suggest superior learning associated with anodal tDCS applied to M1 contralateral to the trained hand (Nitsche et al., 2003b; Kang and Paik, 2011). Thus, it comes as a surprise that in the present data left hand performance was attenuated, although the right M1 received anodal tDCS. This result may support the relevance of the contralateral (left) over the ipsilateral (right) M1 for motor learning. Performance changes of the untrained hand argue against the possibility that unilateral training may equally involve bilateral M1. As a consequence, the gain of reaction times of the untrained left hand did not exceed that of the trained right hand, although the excitability of the contralateral (right) M1 was increased due to anodal tDCS. We realize that this interpretation is speculative at the moment and that the data raise the question for the effects of anodal tDCS applied to the left M1 concurrently to training on the SRTT with the left hand.

In line with our hypothesis, motor sequence acquisition was significantly reduced by anodal tDCS and this result is in line with a recent study by Kobayashi et al. (2009) showing that reducing left M1 excitability by means of 1 Hz rTMS facilitated motor sequence learning of the ipsilateral hand. As suggested by these authors, one possible explanation for the effects of NIBS on performance of the ipsilateral hand might be an alteration of the interhemispheric interaction between bilateral M1. Normally, the excitability of both hemispheres is delicately balanced (Hilgetag et al., 2001). Reducing the excitability of one hemisphere by 1 Hz rTMS, may lead to increased activity of the non-stimulated hemisphere (Hilgetag et al., 2001; Kobayashi et al., 2004). Using NIBS, it has been shown that the unilateral alteration of M1 excitability modulates activity and metabolic rates in the contralateral homologous area (Strafella and Paus, 2001; Chouinard et al., 2003; Schambra et al., 2003; Kobayashi et al., 2004; Lang et al., 2005; Stagg et al., 2009) as well as the interhemispheric inhibition (IHI) from the stimulated to the unstimulated M1 (Gilio et al., 2003; Pal et al., 2005; Tazoe et al., 2014). Given that the interaction between bilateral M1 is predominantly inhibitory (Ferbert et al., 1992), increasing the activity in one hemisphere is presumably associated with more pronounced inhibition onto the other one. In the present study anodal tDCS was applied 8 min before and concurrently with the SRTT training. Thus, the right M1 may have exerted a stronger inhibitory effect onto the left M1 by the time training had started, thereby hampering sequence acquisition with the right hand. There is indeed evidence that anodal tDCS applied to the non-dominant (right) M1 increases IHI from the right to the left M1 (Tazoe et al., 2014). The present result is in line with findings showing that tDCS does not only affect the cortical area under the stimulation electrode but may additionally modulate remote interconnected neuronal networks (Boros et al., 2008; Polania et al., 2011) including M1 when stimulating its contralateral homolog (Lang et al., 2005; Stagg et al., 2009).

An alternative explanation for the attenuation of performance following anodal tDCS could be that this effect was causally related to a modulation of activity in the ipsilateral, right M1. While involvement of the right M1 has been observed to some extent, the left M1 is predominantly involved in motor sequence learning of either hand in right-handers (Karni et al., 1995; Hazeltine et al., 1997; Honda et al., 1998; Grafton et al., 2002). But, in contrast to contralateral crossed corticospinal projections, ipsilateral, uncrossed corticospinal pathways are sparse (Nathan et al., 1990). Therefore, it seems less likely that the observed effect was due to an activation of the ipsilateral motor pathway.

The present findings support the hypothesis of interhemispheric rivalry. Its possible clinical relevance has been indicated in patients suffering from focal hand dystonia (Furuya et al., 2014) as well as in stroke patients (Takeuchi et al., 2012). The data by Furuya and co-workers nicely suggest that increasing the excitability of the unaffected hemisphere by means of tDCS may yield improved task performance of the affected hand but only when the excitability of the affected hemisphere was concurrently reduced. Either reducing the excitability of the affected hemisphere or increasing the excitability of the unaffected side, did not significantly modulate task performance. In the study by Takeuchi et al. (2012) the combination of anodal tDCS applied to the affected M1 and low-frequency rTMS applied to the unaffected homolog changed the transcallosal inhibition balance of both hemispheres in stroke patients. Although those data suggest that the combination of NIBS-protocols differentially affecting the excitability of either hemisphere may have the potential to facilitate rehabilitation, the findings raise the question whether alterations of the affected hemisphere spontaneously activate (in case of stroke) or down-regulate (in case of hyper-activation) the respective homolog area. A number of studies investigated the effect of excitability changes of one hemisphere on that of the homolog area in stroke patients and found evidence for contralesional hyperactivation (e.g., Nelles et al., 1999; Favre et al., 2014; Volz et al., 2015). But, the functional significance of the observed changes is still a matter of debate. While some studies suggest a functional role of the unaffected hemisphere for recovery (Nelles et al., 1999; Volz et al., 2015), others found no significant effect (Favre et al., 2014). Even evidence for a detrimental role of enhanced negative coupling between bilateral M1 was found (Marshall et al., 2009). The data by Volz et al. (2015) support the assumption of contralesional hyperactivation and suggest that this may occur due to reduced transcallosal inhibition exerted by the affected hemisphere (Volz et al., 2015). Those data indeed support the hypotheses (*i*) that alterations within one hemisphere yields changes of interhemispheric connectivity (*ii*) that such changes contribute to the amount of motor impairment and impact on recovery after stroke. Using TMS Swayne et al. (2008) showed alterations of intra-cortical excitability of both hemispheres occurring already in the early post-stroke period persisting up to 6 months. The fact that changes within the unaffected hemisphere occur early after stroke reveal a piece of evidence for the assumption that they may occur spontaneously due to alterations of the affected side. Nevertheless, we would like to stress that to the best of our knowledge no direct evidence for this assumption exists. Noteworthy, the functional contribution of such changes varies over the course of the post-stroke period suggesting a significant correlation with hand functions after a time period of about 3 months but not in the early post-stroke period (Swayne et al., 2008).

Taken together, the present data provide evidence for an inhibitory effect of anodal tDCS applied to the right M1 on implicit motor sequence learning of both hands. The underlying neurophysiological mechanisms likely involve alterations of the interaction between bilateral M1 supporting the hypothesis of interhemispheric rivalry. The present findings in combination with those from patient studies suggest the benefit of NIBS protocols that differentially affect the excitability of bilateral M1 for neurorehabilitation. Nevertheless, the exact underlying mechanisms – in particular the question whether alterations within one hemisphere spontaneously yield excitability changes in homolog areas – remains speculative.

## Author Contributions

BP, VK, and AK planned and designed the experiments. HØ and AK acquired and analyzed the data. BP, VK, AK, and HØ interpreted the data and critically reviewed and revised the manuscript. AK and BP wrote the manuscript.

## Conflict of Interest Statement

The authors declare that the research was conducted in the absence of any commercial or financial relationships that could be construed as a potential conflict of interest.
